# Sacituzumab Tirumotecan Across Gynecologic Malignancies: One Target, Multiple Diseases

**DOI:** 10.1007/s11912-026-01806-2

**Published:** 2026-06-22

**Authors:** Amalia A. Sofianidi, Evangelia Panagodimou, Emmanouil Kalampokas, Ioannis Georgakopoulos, Flora Zagouri

**Affiliations:** 1 Oncology Unit, Aretaieion University Hospital, National and Kapodistrian University of Athens School of Medicine, Vasilissis Sofias 76, Athens, 115 28 Greece; 2 Second Department of Obstetrics and Gynecology, Aretaieion University Hospital, National and Kapodistrian University of Athens, Athens, 11527 Greece; 3 Radiation Oncology Unit, First Department of Radiology, Medical School, Aretaieion Hospital, National and Kapodistrian University of Athens, 11527 Athens, Greece

**Keywords:** Sacituzumab tirumotecan, Sac-TMT, MK-2870, Cervical, Ovarian, Endometrial, Antibody drug conjugates

## Abstract

**Purpose of Review:**

Sacizuzumab Tirumotecan is a novel antibody-drug conjugate targeting TROP2 glycoprotein, which is highly expressed across malignant cells. Sacizuzumab Tirumotecan has already demonstrated promising clinical activity in combating breast and lung cancer, and it is currently under evaluation for use against gynecologic malignancies. This review aims to highlight the therapeutic potential of Sacituzumab Govitecan in gynecologic malignancies and to summarize the most recent evidence supporting its clinical application.

**Recent Findings:**

Ongoing phase III trials of Sacituzumab Tirumotecan include cervical, ovarian, and endometrial cancers. Preliminary safety data of Sacituzumab Tirumotecan indicates a manageable toxicity profile, with the most commonly reported adverse events including hematologic toxicity, oral mucositis, and alopecia.

**Summary:**

Mature data from Phase III trials are anticipated to clarify the impact of Sacituzumab Tirumotecan on overall survival, progression-free survival, and objective response rates. Additionally, biomarker analyses may enhance patient selection strategies and refine prognostic assessment in gynecologic oncology.

## Highlights

Antibody-drug conjugates (ADCs) are an advent in the management of oncology patients.

Sacituzumab Tirumotecan (sac-TMT/MK-2870/SKB264) is a promising new ADC undergoing clinical trials in solid tumors.

Promising results have been published with the use of Sacituzumab Tirumotecan in gynecologic malignancies, mainly cervical, ovarian and endometrial cancers.

More mature data from phase III trials are expected soon.

## Introduction

Gynecological cancers pose a significant global health burden, with increasing trends in the past few years [1, 2]. Cervical, ovarian, and endometrial cancers are the most frequently diagnosed gynecological malignancies and constitute more than one-third of the newly diagnosed cancers globally in females. A total of 1,473,427 women were diagnosed with gynecological cancer in 2022, with 44.95% of these cases being identified as cervical cancer [2, 3]. Indeed, cervical cancer ranks highly regarding incidence and mortality, with a 5-year survival rate of 67% across all stages of the disease [4]. Patients diagnosed with ovarian cancer have an even worse prognosis, with 51% of patients remaining alive 5 years after diagnosis [5]. Endometrial cancer appears to have the most favorable prognosis of all, with a 5-year survival rate of 84% when all stages of the disease are combined [6]. However, outcomes remain particularly poor for patients diagnosed with advanced or recurrent disease, especially those with distant metastases. This is reflected in markedly lower 5-year survival rates in metastatic settings: approximately 19% for cervical cancer, 32% for ovarian cancer, and 22% for endometrial cancer [4–6].

Cytotoxic chemotherapy is not the only weapon we have in gynecologic oncology. Immunotherapy and various forms of targeted therapy have been implemented in clinical practice during the past few years [7, 8]. However, in the twenty-first century, when massive increases in life expectancy have occurred, novel therapeutic modalities are urgently needed to increase the life expectancy of patients diagnosed with gynecologic malignancies, even at an advanced stage. Antibody-drug conjugates (ADCs) have reshaped the therapeutic landscape of oncology. By combining the effects of both cytotoxic chemotherapy and targeted therapy, ADCs deliver potent cytotoxic agents to malignant cells that express a specific cell surface marker [9]. To date, the only ADCs approved for use in gynecologic oncology are Tisotumab Vedotin and Mirvetuximab Soravtasine. Tisotumab Vedotin targets tissue factor (TF), and both the US Food and Drug Administration (FDA) and European Medicines Agency (EMA) have approved it for use as second- or third-line treatment option in patients with recurrent cervical cancer [8]. Mirvetuximab soravtansine is a novel ADC targeting folate receptor α (FRα) that has been approved by the FDA and EMA for the treatment of adult patients with FRα-positive, platinum-resistant epithelial ovarian, fallopian tube, or primary peritoneal cancer following one to three prior systemic therapies [10]. Potential new targets are being investigated, and trophoblast cell-surface antigen 2 (TROP2) has emerged as a valuable candidate.

TROP2 is a 36-kDa cell surface glycoprotein encoded by the tumor-associated calcium signal transducer 2 (*TACSTD2)* gene, playing a vital role in intracellular calcium signaling, cell cycle regulation, and proliferation [11]. TROP2 is expressed along several, mostly epithelial, normal tissues; however, the overexpression of this protein has been observed in various human carcinomas [11]. Data extracted from the Human Protein Atlas shows that TROP2 is highly expressed in gynecologic cancer tissues, primarily in cervical carcinoma, as well as in malignant ovarian and endometrial cells [12]. The specific expression of TROP2 in gynecological malignancies identifies it as a promising candidate for ADC development.

Sacituzumab Tirumotecan (sac-TMT, also known as SKB264 or MK-2870) is an innovative TROP2-directed ADC consisting of a recombinant humanized monoclonal antibody (mAb) that targets TROP2 glycoprotein, a novel linker, 2-methylsulfonyl pyrimidine, and a belotecan-derivative topoisomerase I inhibitor that acts as the payload [13]. This ADC is innovative because it integrates a novel linker designed to improve bioactivity and a wider therapeutic index, with milder off-target effects [13]. Sacituzumab Tirumotecan uses a sulfonyl-pyrimidine CL2A-carbonate linker conjugating a belotecan-derived topoisomerase I payload at a drug-to-antibody ratio ≈ 7.4, which is close to the maximum drug-to-antibody ratio achievable via standard cysteine conjugation [13]. This linker allows pH-sensitive and enzymatic cleavage, making Sacituzumab Tirumotecan a more stable ADC and limiting off-target toxicity [13]. Sacituzumab tirumotecan has demonstrated mature and clinically meaningful activity in other tumor types, including breast [14] and lung cancers [15] and is currently under investigation in gynecologic malignancies. The aim of this review is to highlight recent advances in the development and application of Sacituzumab Tirumotecan across the most frequently diagnosed gynecologic malignancies, namely cervical, ovarian, and endometrial cancers.

## Sacituzumab Tirumotecan in Gynecologic Malignancies

### Sacituzumab Tirumotecan in Cervical Cancer

Τhe prognosis of patients with recurrent or metastatic cervical cancer remain extremely poor, with limited treatment options and a median overall survival that rarely exceeds 12–18 months [16]. The biological rationale for evaluating Sacituzumab Tirumotecan in cervical cancer is supported by high expression levels of TROP2 protein across squamous cell and adenocarcinoma histologies [17]. Approximately 90% of cervical cancer cases demonstrate intense membranous staining of TROP2. Squamous cell carcinomas demonstrate higher levels of TROP2 expression compared to adenocarcinomas (*p* = 0.023). Additionally, a greater proportion of squamous tumors exhibit moderate to strong TROP2 staining [18]. Notably, strong TROP2-expression is associated with poor prognosis in cervical carcinoma patients [19]. First-line treatment for metastatic cervical carcinoma consists of platinum-based chemotherapy with or without the antiangiogenetic agent bevacizumab, combined with the immunotherapeutic agent pembrolizumab [20]. With a median progression-free survival of 10.4 months, subsequent therapeutic options remain limited [20]. It was only recently that Tisotumab Vedotin was added to the therapeutic landscape for recurrent or metastatic cervical carcinoma, with a median progression-free survival of 4.2 months, as compared to 2.9 months with standard chemotherapy in pretreated patients. These results do not prolong life expectancy much. The phase I/II innovaTV 205/GOG-3024/ENGOT-cx8 study showed preliminary results of TV in combination with bevacizumab, carboplatin, or pembrolizumab that might result in better outcomes [21]. Nevertheless, new therapeutic options are needed, and a TROP2-directed ADC could represent a promising addition to this treatment landscape.

Following the addition of pembrolizumab to first-line treatment options for patients with metastatic cervical cancer and given its strong survival benefit for this population, attempts are being made to combine pembrolizumab with Sacituzumab Tirumotecan. The rationale for combining immunotherapy with this TROP2-targeting ADC is based on the fact that TROP2 expression is correlated with immune-infiltrating tumor microenvironments (TMEs) [17]. Researchers recently found that TROP2 expression is positively correlated with intratumoral CD3+/CD8 + tumor-infiltrating lymphocytes (TILs) in cervical cancer, suggesting the combination of TROP2-targeted therapy and immune checkpoint inhibitors (ICIs) [17]. Mounting evidence suggests that ADCs can increase the efficacy of immunotherapeutic agents [22]. The initiation of immunogenic cell death, increased T lymphocyte infiltration; the potentiation of immunological memory; and the expression of immune-regulatory proteins, such as programmed-death ligand 1 (PD-L1), are among the potential mechanisms [23–25]. A Phase II basket study (NCT05642780) is currently underway to assess the efficacy of Sacituzumab Tirumotecan in combination with pembrolizumab across a range of tumor types, including cervical cancer. In a cervical cancer cohort, Sacituzumab Tirumotecan plus pembrolizumab demonstrated promising and durable antitumor activity as a second- or third-line option in patients with recurrent or metastatic cervical cancer, though these results were preliminary [26]. At a median follow-up of 6.2 months, the objective response rate was 57.9%, with 50.0% of responses being confirmed [26]. The 6-month duration of response rate was 82.1% [26]. Median progression-free survival was not reached, and the 6-month progression-free survival rate was 65.7% [26]. Encouraging clinical activity was observed regardless of tumor PD-L1 status and prior anti-programmed death-1 (PD-1) therapy [26]. Overall, Sacituzumab Tirumotecan combined with pembrolizumab demonstrated a manageable safety profile, with hematologic toxicity being the most reported treatment-related adverse event [26]. One patient (2.6%) reported grade-2 interstitial lung disease but successfully recovered [26]. More mature data are expected in the following years. Notably, a Phase III study has been initiated (MK-2870-036/TroFuse-036/GOG-3123/ENGOT-cx22) investigating the addition of Sacituzumab Tirumotecan to pembrolizumab as a maintenance treatment in patients with metastatic cervical cancer.

Sacituzumab Tirumotecan is also currently being investigated as a monotherapy option in advanced or metastatic cervical cancer. Promising antitumor activity was reported in a Phase I/II clinical trial (MK-2870-001/KL264-01, NCT04152499) of Sacituzumab Tirumotecan monotherapy in pretreated cervical cancer patients [27]. The primary endpoint, objective response rate, was reported to be 28% [95% confidence interval (CI), 17–41], and the median progression-free survival was 6.1 months (95% CI, 3.9–NE) [27]. Hematologic toxicity was, again, the most frequent treatment-related adverse event, including Grades 3 and 4 [27]. One patient discontinued treatment because of Grade-3 anemia [27]. These data led to the initiation of the ongoing Phase III trial GOG-3101/ENGOT-cx20 (TroFuse-020; NCT06459180), which compares Sacituzumab Tirumotecan with standard chemotherapy or Tisotumab Vedotin in patients with recurrent or metastatic cervical cancer. The study has completed enrollment, and results are pending maturation of the required events for the final analysis [28].

Table 1 summarizes the clinical trials of Sacituzumab Tirumotecan in cervical cancer.

**Table 1:** Clinical trials of Sacituzumab Tirumotecan in cervical cancer

Clinical trial ID	Phase	Disease	Drugs	Pts	Status	Preliminary results	TRAEs
NCT05642780	II	Cohort A: Subjects with recurrent or metastatic cervical cancer	Sac-TMT + Pembrolizumab	240(all cohorts)	Active, not recruiting	mFU 6.2 mo; ORR 57.9% (50% confirmed); 6-mo DOR 82.1%; mPFS NR; 6-mo PFS 65.7%.	Grade 2 ILD: 1 pt (2.6%) TRAEs Grade ≥ 3: neutrophil count decreased 23.7%, anemia 21.1%, WBC decreased 15.8%, Lymphocyte count decreased 10.5%, stomatitis 5.3% [26].
MK-2870-001/KL264-01 (NCT04152499)	I/II	Advanced/metastatic cervical cancer	Sac-TMT monotherapy	1410 (all cohorts)	Active, not recruiting	Data cutoff (Nov 18, 2024), 58 pts treated: mFU 6.8 mo; ORR 66% (28% confirmed); 6-mo DOR NR; mPFS 6.1 mo;	TRAEs Grade ≥ 3: anemia 26%, neutrophil count decreased 14%, WBC decreased 12% [27].
MK-2870-036/TroFuse-036/GOG-3123/ENGOT-cx22 (NCT07216703)	III	metastatic cervical cancer	Sac-TMT + Pembrolizumab +/- Bevacizumab vs. Pembrolizumab +/- Bevacizumab (1st line maintenance treatment)	1023	Recruiting	n/a	n/a
GOG-3101/ENGOT-cx20 /TroFuse-020 (NCT06459180)	III	metastatic cervical cancer	Sac-TMT monotherapy vs. TPC (2nd line treatment)	686	Recruiting	n/a	n/a

*DOR *duration of response, *ILD *interstitial lung disease, *mFU *median follow-up, *mPFS *median progression-free survival, *NR *not reached, *ORR *objective response rate, *PFS *progression-free survival, *pt(s) *patient(s), *Sac-TMT *Sacituzumab Tirumotecan, *TPS *Treatment of Physician’s Choice, *TRAEs *treatment related adverse events, *Vs *versus, *WBC *white blood cells

### Sacituzumab Tirumotecan in Ovarian Cancer

Advanced ovarian cancer has poor survival rates, which is attributed both to late diagnosis and the lack of effective second-line therapy for patients who relapse. 32% of patients with metastatic ovarian cancer are alive 5 years after diagnosis [5]. The development of platinum resistance is associated with poorer clinical outcomes; the median overall survival of this subset of patients is approximately 12–18 months [29]. It was only recently that a novel ADC, Mirvetuximab Soravtasine, targeting FRa, a biomarker that is commonly overexpressed in ovarian carcinomas [30] and minimally expressed in normal tissues, was approved in this setting [10]. Indeed, approximately 60% of ovarian cancers express FRα, with reported expression rates ranging from 75 to 85% in high-grade serous tumors [30]. Treatment with Mirvetuximab Soravtasine resulted in a median progression-free survival of 5.62 months; however subsequent therapeutic options are scarce and rely on standard chemotherapy regimens that yield only modest results [10].

Moderate to strong TROP2 expression has been observed in 80.8% of endometrioid endometrial carcinomas, 74.5% of serous carcinomas, 66.7% of clear cell carcinomas, and 52.4% of uterine carcinosarcomas [31]. The Phase I/II study (MK-2870-001/KL264-01, NCT04152499) of Sacituzumab Tirumotecan in patients with solid tumors that were refractory to standard treatments enrolled patients with advanced ovarian cancer as well. Preliminary results in this cohort showed that among 40 patients with advanced ovarian cancer who were heavily pretreated, the objective response rate was 40%, and the disease control rate was 75%. At a median follow-up of 28.2 months, median progression-free survival was 6.0 months, and median overall survival was 16.5 months. Importantly, the focus was platinum-resistant ovarian cancer; 87.5% of patients enrolled were platinum resistant, yielding an median progression-free survival of 6.0 months and an median overall survival of 16.1 months [32]. The most frequently reported treatment-related adverse events were hematologic in nature, with a higher incidence of Grade ≥ 3 events. The most common gastrointestinal toxicities were stomatitis, vomiting, and nausea [32]. Stomatitis, a well-described off-target toxicity caused by ADCs [33], has been observed in 6 patients at a grade greater than 3 [32]. Notably, no treatment-related adverse events led to death, and no incidences of interstitial lung disease or pneumonitis were described. These promising results may suggest new therapeutic modalities for patients with ovarian cancer that is refractory to platinum-based regimens, which is a population that is difficult to treat.

While the focus is mainly on platinum-refractory disease, efforts are being made to improve outcomes in platinum-sensitive disease, which tends to lead to relapse. After achieving a complete response with first-line platinum-based chemotherapy, > 70% of the patients with ovarian cancer experience relapse at 2 years [34]. Rechallenge with platinum-based regimens is the key, but eventually, the disease tends to become refractory to platinum-based regimens [34]. A Phase III trial (MK-2870-022/TroFuse-022/ENGOT-ov84/GOG-3103, NCT06824467) is currently active, enrolling patients with platinum-sensitive ovarian cancer. Patients enrolled must have received two prior lines of platinum-based systemic therapy before starting the investigational maintenance therapy. The primary focus of this trial is to study whether maintenance treatment with Sacituzumab Tirumotecan monotherapy or Sacituzumab Tirumotecan in combination with bevacizumab is superior to the standard of care, bevacizumab, in terms of efficacy, safety, and quality of life improvement. In parallel, the Phase III randomized, open-label, multicenter TroFuse-021/ENGOT-ov85/GOG-3102 study opened for enrollment recently and will evaluate maintenance Sacituzumab Tirumotecan, with or without bevacizumab, versus standard of care in patients with newly diagnosed advanced homologous recombination deficiency (HRD)-negative ovarian cancer following first-line platinum-based chemotherapy.

Table 2 summarizes the clinical trials of Sacituzumab Tirumotecan in ovarian cancer.

**Table 2:** Clinical trials of Sacituzumab Tirumotecan in ovarian cancer

Clinical Trial ID	Phase	Disease	Drugs	Pts	Status	Preliminary Results	TRAEs
MK-2870-001/KL264-01 (NCT04152499)	I/II	Advanced/metastatic ovarian cancer	Sac-TMT monotherapy	1410 (all cohorts)	Active, not recruiting	Data cutoff (Mar 5, 2024), 40 pts treated: mFU 28.2 mo; ORR 40% (35% confirmed); mOS 16.5 mo; mPFS 6.0 mo;	TRAEs Grade ≥ 3: WBC decreased 22.5%, anemia 14%, neutrophil count decreased 12%, stomatitis 15% [32].
MK-2870-022/TroFuse-022/ENGOT-ov84/GOG-3103 (NCT06824467)	III	Platinum sensitive ovarian cancer	Sac-TMT monotherapy vs. Sac-TMT + Bevacizumab (maintenance treatment after two prior lines of platinum-based systemic therapy)	770	Recruiting	n/a	n/a
TroFuse-021/ENGOT-ov85/GOG-3102 (NCT07318558)	III	newly diagnosed advanced HRD-negative ovarian cancer	Sac-TMT +/-Bevacizumab vs. SoC (maintenance after 1st line platinum-based chemo)	900	Recruiting	n/a	n/a

*Chemo *chemotherapy, *HRD* Homologous recombination deficiency, *mFU *median follow-up, *mo *months, *mOS *median overall survival, *mPFS *median progression-free survival, *ORR *objective response rate, *pts *patients, *Sac-TMT *Sacituzumab Tirumotecan, *SoC *standard of care, *TRAEs *treatment related adverse events, *Vs *versus, *WBC *white blood cells

### Sacituzumab Tirumotecan in Endometrial Cancer

TROP2 protein is highly expressed in many endometrial carcinomas [35], especially poorly differentiated endometrioid carcinoma [36] and uterine carcinosarcoma [37]. 65% to 95% of uterine serous carcinoma cases express TROP2, with 60% of cases reported as moderate to strong expressors in one report [36, 38]. Additionally, TROP2 expression has been observed in 62.5% of uterine carcinosarcoma cases [39]. TROP2 has been characterized as a poor prognostic factor for the disease, being associated with worse overall survival rates [40]. The prognosis of patients with advanced or recurrent endometrial cancer are poor; First-line treatment is currently guided by the results of the RUBY and NRG-GY018 trials, which demonstrated that the addition of an anti-PD-1 monoclonal antibody (dostarlimab or pembrolizumab) to carboplatin and paclitaxel improves outcomes, establishing chemoimmunotherapy as the standard of care [7, 41]. For patients who have been previously treated with platinum-based chemotherapy and immunotherapy, subsequent treatment options are limited. The results of a Phase I/II clinical trial (MK-2870-001/KL264-01, NCT04152499) evaluating Sacituzumab Tirumotecan monotherapy in patients with advanced unresectable or metastatic solid tumors, including endometrial cancer, who were refractory to the available standard therapies demonstrated promising clinical activity. In the endometrial cancer cohort, confirmed objective response rates were 29.8% and 34.1% in the 4 mg/kg and 5 mg/kg groups, respectively, with all responses being partial responses. Median progression-free survival was 5.6 and 7.3 months, respectively, while median duration of response was not reached in the 4 mg/kg cohort and was 7.6 months in the 5 mg/kg cohort. Notably, activity was observed in heavily pretreated patients, including those previously exposed to ICIs, and the safety profile was manageable. Based on these findings, the 4 mg/kg every 2 week regimen was selected for further Phase III development [42]. Safety data were consistent with those for the ovarian cohort [32]. Furthermore, while no Phase III efficacy or safety results are yet available for TROP2-directed ADCs in endometrial cancer, the Phase III ENGOT-en23/GOG-3095/MK-2870-005 trial, comparing Sacituzumab Tirumotecan monotherapy with chemotherapy in patients with previously treated disease (including prior chemotherapy and/or anti-PD-(L)1 therapy), has completed enrollment, and results are awaited pending maturation of the required events for final analysis [43]. Patients with histologically confirmed carcinosarcoma are also eligible, representing a population with even worse survival outcomes [44].

Table 3 depicts the clinical trials of Sacituzumab Tirumotecan in endometrial cancer.

**Table 3:** Clinical trials of Sacituzumab Tirumotecan in endometrial cancer

Clinical trial ID	Phase	Disease	Drugs	Pts	Status	Preliminary results	TRAEs
MK-2870-001/KL264-01 (NCT04152499)	I/II	Advanced/metastatic endometrial cancer	Sac-TMT monotherapy	1410 (all cohorts)	Active, not recruiting	Data cutoff (Mar 5, 2024), 44 pts treated: mFU 7.2 mo; ORR 34.1% (27.3% confirmed); mPFS 5.7 mo;	TRAEs Grade ≥ 3: neutrophil count decreased 43.2%, WBC decreased 40.9%, anemia 29.5%, stomatitis 13.6% [32].
ENGOT-en23/GOG-3095/MK-2870-005 (NCT06132958)	III	Advancedendometrial cancer previously treated with platinum-based chemo and immunotherapy	Sac-TMT monotherapy vs. TPC	710	Active, not recruiting	n/a	n/a

*Chemo *chemotherapy, *mFU *median follow-up, *mPFS *median progression-free survival, *mo *months, *ORR *objective response rate, *Pts *patients, *Sac-TMT* Sacituzumab Tirumotecan, *TPS *Treatment of Physician’s Choice, *TRAEs *treatment related adverse events, *Vs *versus, *WBC *white blood cells

## Sacituzumab Tirumotecan Versus Other TROP2-Targeting ADCs

It is beyond doubt that TROP2 is a promising target in solid tumors. Several TROP2-targeting ADCs are currently undergoing evaluation in clinical trials for use with several solid tumors. In addition to the well-described Sacituzumab Govitecan, newly described TROP2-targeting ADCs, such as Sacituzumab Tirumotecan and Datopotamab Deruxtecan have been launched and are being investigated in gynecologic malignancies [45]. All three TROP2-targeting ADCs have topoisomerase I inhibitors as payloads; KL610023 for Sacituzumab Tirumotecan [13], SN-38 for Sacituzumab Govitecan [46], and Dxd for Datopotamab Deruxtecan [47]. Sacituzumab Govitecan has a high drug-to-antibody ratio of 7.6 [46], which is comparable to the 7.4 of Sacituzumab Tirumotecan. However, Datopotamab Deruxtecan has a drug-to-antibody ratio of approximately 4 [48], which is considered low as less payload is delivered to the tissues compared to other TROP2-targeting ADCs; nevertheless, this drug design holds the potential to mitigate toxicity. Sacituzumab Tirumotecan was dosed at 5 mg/kg every two weeks, as compared to Datopotamab Deruxtecan’s schedule of 6 mg/kg every 3 weeks [32, 49]. Sacituzumab govitecan dose schedule is 10 mg/kg on day 1 and 8 of each 21-day cycle [50].

Both Sacituzumab Govitecan and Datopotamab Deruxtecan are currently under evaluation in phase III trials, enrolling patients with gynecologic malignancies. Preliminary data from Phase II trials have been published. In the Phase II TROPiCS-03 study (NCT03964727), patients with advanced endometrial cancer who had received a median of three prior lines of therapy achieved a median duration of response of 8.8 months and a median progression-free survival of 4.8 months [51]. In the Phase II TROPION-PanTumor03 study (NCT05489211), Datopotamab Deruxtecan was evaluated as a monotherapy option in heavily pretreated ovarian and endometrial cancer patients. In the endometrial cancer cohort, an objective response rate of 27.5%, a median progression-free survival of 6.3 months, and a median duration of response of 16.4 months were found. In the ovarian cancer cohort, the objective response rate was 42.9%, the median duration of response was 5.7 months, and the median progression-free survival was 5.6 months [49].

The safety profile and the adverse events of Sacituzumab Govitecan have been well described though its use in breast malignancies. Hematologic toxicity, nausea and diarrhea are its major reported adverse events [52]. Hematologic toxicity does not appear to be a concern with Datopotamab Deruxtecan. The most common treatment-related adverse events of this ADC included any grade of stomatitis and nausea. However, when discussing the safety profile of Datopotamab Deruxtecan, it would be important to specifically highlight ocular surface events and ILD, as these represent adverse events of special interest for this agent class. Ocular events have occurred in a clinically meaningful proportion of patients (up to 40% in some cohorts, predominantly grade ≤ 2) [53], and drug-related interstitial lung disease (ILD) cases, including grade 3, were also reported [49].

Sacituzumab Tirumotecan has demonstrated an acceptable safety profile across clinical trials. Hematologic toxicity of Grade 3 or higher is the most frequent serious treatment-related adverse event, although it is manageable with the supportive use of growth factors [15]. Alopecia is a major concern with this ADC [32], which is consistent with findings reported for other ADCs [54], including Datopotamab Deruxtecan [49]. Stomatitis, a well-described on-target, off-tumor toxicity caused by ADCs, is also observed with Sacituzumab Tirumotecan. It should be noted that preventative measures are implemented, making drug discontinuation due to this event scarce [15]. No drug-related events of interstitial lung disease or pneumonitis have been reported [32]. Comparisons between these three ADCs should be interpreted with caution given the immaturity of the currently available data. Although all three agents have demonstrated antitumor activity in gynecologic malignancies, definitive conclusions await the availability of mature results from ongoing Phase III trials.

**Fig. 1:**
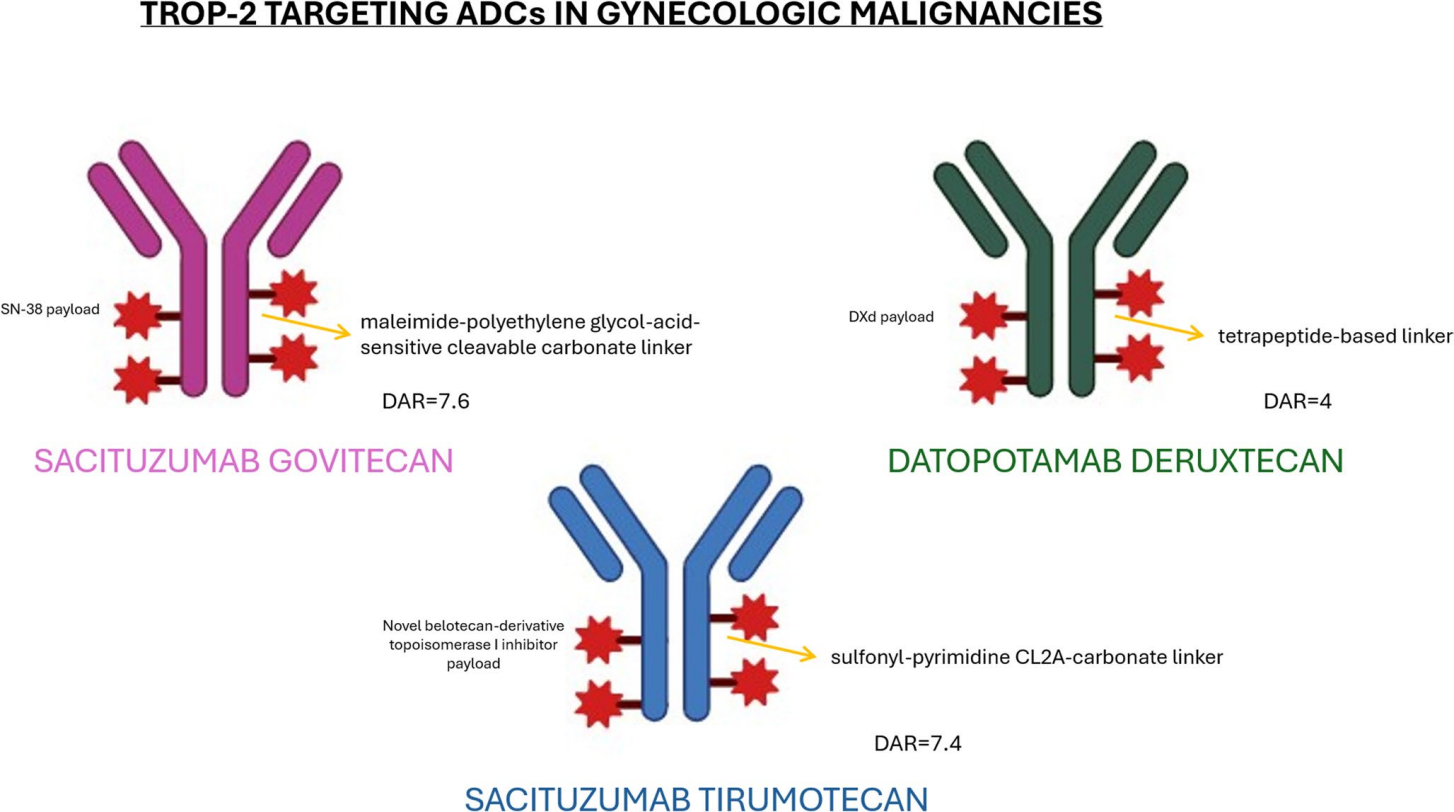
TROP-2 targeting ADCs undergoing evaluation in gynecologic malignancies. ADCs=antibody-drug conjugates, DAR=drug-to-antibody ratio, DXd=deruxtecan

Figure 1 depicts the three different TROP-2 targeting ADCs undergoing evaluation in gynecologic malignancies.

## Conclusion-Future Perspectives

One interesting avenue for future research is the potential use of TROP2 expression as a stratification factor for administering TROP2-targeted ADCs. There are no standardized protocols and interpretation criteria for TROP2 expression available. Tumor cell membrane TROP2 expression is currently assessed using a histochemical score (H-score), which combines the intensity of staining with the proportion of positively stained cells to generate a weighted numerical value. H-scores are calculated using the following formula: H-score = (3 × % cells with strong intensity staining) + (2 × % cells with moderate intensity staining) + (1 × % cells with mild intensity staining). Subsequently, categories are created based on the distribution of the H-score (range from 0 to 300) to divide the population into low, medium, and high expression subgroups. TROP2 expression is classified into three categories based on H-score: 0 to < 100 as low, 100–200 as medium, and > 200–300 as high [55]. In the endometrial cancer cohort of the Sacituzumab Tirumotecan Phase I/II trial (MK-2870-001/KL264-01, NCT04152499), among patients with TROP2 immunohistochemistry (IHC) H-scores > 200 (*n* = 12) or ≤ 200 (*n* = 28), the objective response rates were 41.7% (5/12; three confirmed responses) and 35.7% (10/28; nine confirmed responses), respectively [32]. Apparently, the objective response rate was numerically higher in patients with TROP2 IHC H-scores > 200 as compared with those with scores ≤ 200; however, clinically meaningful responses were observed in both subgroups, suggesting that Sacituzumab Tirumotecan activity in endometrial cancer is not restricted to tumors with high TROP2 expression. Data on TROP2-targeting ADCs in lung cancer do not show a strong correlation between TROP2 IHC level and clinical benefit, suggesting that TROP2 presence is required in cancerous tissue but that it is not a good quantitative biomarker for treatment efficacy [56, 57]. Similar observations were made in breast cancer trials of Sacituzumab Tirumotecan; triple-negative breast cancer patients with high and low tissue TROP2 expression achieved comparable progression-free survival outcomes [58]. Rather than relying on bulk TROP2 expression, a more nuanced understanding is recommended, that incorporates spatial drug distribution [59], TROP2 endocytosis biology [60], and genomic context [61] as the true determinants of TROP2 ADC efficacy. Recently, assays with greater sensitivity and less subjectivity than H-score have been also developed, namely quantitative immunofluorescence (QIF) and quantitative hematoxylin-DAB (QH-DAB) assays, which may eventually serve as companion diagnostics if clinical validation confirms a meaningful TROP2 expression threshold [62]. Future trials could focus on the dynamic assessment of TROP2 expression during treatment to clarify mechanisms of response and resistance.

Combination strategies involving Sacituzumab Tirumotecan also represent a promising area of research and warrant further exploration. The most promising strategy to date is the combination of Sacituzumab Tirumotecan with immunotherapy, with the most recent data implying that Sacituzumab Tirumotecan could enhance anti-tumor immunity, creating synergy with ICIs that cannot be achieved by either agent alone. A 2026 study demonstrated that TROP2 is associated with claudin-7, regulating tight junction formation and thus, creating a physical barrier that prevents T-cell infiltration. This means the anti-TROP2 antibody component of Sacituzumab Tirumotecan may directly convert immune-excluded (“cold”) tumors into immune-infiltrated (“hot”) tumors amenable to checkpoint blockade [63]. This approach is being evaluated across gynecologic malignancies, with mature clinical data anticipated soon.In conclusion, Sacituzumab Tirumotecan is a novel TROP2-targeting ADC, currently under evaluation across various gynecological tumors, with the scientific focus being oriented toward cervical, ovarian, and endometrial cancers. Sacituzumab Tirumotecan has shown more mature positive signals in other solid tumor types, such as breast and lung cancers, which drives enthusiasm and investment but also increases the risk of overextrapolation to gynecologic disease without histology-specific evidence. Mature phase III data is anticipated, with a focus on various gynecologic histologies and analyses of efficacy according to TROP2 expression levels. There is no doubt that further research is needed to validate the efficacy of Sacituzumab Tirumotecan across gynecologic malignancies through clinical trials that define not only progression-free survival but also meaningful quality of life data as primary endpoints.

## Key References

- Killock, D. Sacituzumab Tirumotecan Improves OS in mTNBC. Nat. Rev. Clin. Oncol. 2025, 22, 382–382, 10.1038/s41571-025-01022-0.
  - ○ Phase III OptiTROP-Breast01 trial performed in China supports the clinical application of Sacituzumab Tirumotecan in TNBC.
- Fang, W.; Wu, L.; Meng, X.; Yao, Y.; Zuo, W.; Yao, W.; Xie, Y.; Zhang, Y.; Cui, J.; Zhang, Y.; et al. Sacituzumab Tirumotecan in EGFR-TKI–Resistant, EGFR -Mutated Advanced NSCLC. N. Engl. J. Med. 2025, NEJMoa2512071, 10.1056/NEJMoa2512071.
  - ○ Published results from the phase III performed in China show promising efficacy of Sacituzumab Tirumotecan in EGFR-mutated NSCLC.
- Wang, D.; Yao, W.; Wang, S.; Zhang, K.; An, R.; Li, B.; Wang, K.; Li, J.; Wang, F.; Lee, J.-Y.; et al. 1168P Efficacy and Safety of Sacituzumab Tirumotecan (Sac-TMT) Monotherapy in Advanced/Metastatic Cervical Cancer: Results from a Phase I/II Study (MK-2870-001/KL264-01). Ann. Oncol. 2025, 36, S770, 10.1016/j.annonc.2025.08.1804.
  - ○ Preliminary results regarding the efficacy of Sacituzumab Tirumotecan in pretreated cervical cancer from a phase II trial.
- Wang, D.; Wang, K.; An, R.; Yu, G.; Zhang, K.; Wang, D.; Jiang, K.; Gao, Y.; Cheng, Y.; Liu, Y.; et al. 715MO Safety and Efficacy of Sacituzumab Tirumotecan (Sac-TMT) in Patients (Pts) with Previously Treated Advanced Endometrial Carcinoma (EC) and Ovarian Cancer (OC) from a Phase II Study. Ann. Oncol. 2024, 35, S548, doi:10.1016/j.annonc.2024.08.777. 10.1016/j.annonc.2024.08.777
  - ○ Preliminary results regarding the efficacy of Sacituzumab Tirumotecan in pretreated ovarian and endometrial cancer from a phase II trial.

## Data Availability

No datasets were generated or analysed during the current study.

## Glossary

PD-1: Anti-programmed death-1
ADCs: Antibody-drug conjugates
CI: Confidence interval
EMA: European Medicines Agency
HRD: Homologous recombination deficiency
ICIs: Immune checkpoint inhibitors
ILD: Interstitial lung disease
IHC: Immunohistochemistry
mAb: Monoclonal antibody
PD-L1: Programmed-death ligand 1
TF: Tissue factor
TROP2: Trophoblast cell-surface antigen 2
TACSTD2: Tumor-associated calcium signal transducer 2
TMEs: Tumor-associated microenvironments
TILs: Tumor-infiltrating lymphocytes
FDA: US Food and Drug Administration
